# Comparison of the efficacy of treatment with clobetasol propionate or bexarotene in early-stage mycosis fungoides^☆^

**DOI:** 10.1016/j.abd.2024.04.011

**Published:** 2024-12-30

**Authors:** Aslı Aksu Çerman, Pinar Ozdemir Cetinkaya, Birgül Özkesici Kurt, Artun Kırker, İlknur Altunay

**Affiliations:** Dermatology Department, Şişli Hamidiye Etfal Training and Research Hospital, University of Health Sciences, Seyrantepe, İstanbul, Turkey

**Keywords:** Bexarotene, Clobetasol, Lymphoma, T-cell, cutaneous, Mycosis fungoides

## Abstract

**Background:**

There are few studies in the literature comparing the effectiveness of topical treatments in early-stage mycosis fungoides (MF).

**Objectives:**

It was aimed to evaluate the clinical efficacy, side effects and topical treatment compliance with bexarotene or clobetasol propionate in early-stage MF.

**Methods:**

A total of 40 patients with stage IA-IB MF were enrolled in the study. Twenty patients were treated with 1% bexarotene gel and 20 patients were treated with 0.05% clobetasol propionate ointment.

**Results:**

In the bexarotene group, 11 patients (55%) had complete clinical response (CCR) and 5 patients (25%) had partial response (PR) while in the clobetasol propionate group, 10 patients (50%) had CCR and 9 patients (45%) had PR. The median duration of remission was 10.5 months in the bexarotene group and 4 months in the clobetasol propionate group. The remission period was statistically significantly longer in the bexarotene group (p = 0.032). Irritation symptoms were statistically significantly more common in the bexarotene group (p = 0.001).

**Study limitations:**

The limitation of the study was its retrospective design.

**Conclusion:**

Both topical bexarotene and topical clobetasol propionate were found to be effective in MF. Irritation symptoms were more common with topical bexarotene. Moreover, the remission period with topical bexarotene was significantly longer.

## Introduction

Mycosis fungoides (MF) is the most common type of primary cutaneous T-Cell lymphomas (CTCLs). Dermatological manifestations of MF are patches, plaques in the early stages of the disease and tumors, erythrodermas in the advanced stages.^1^ Early lesions are erythematous, scaly patches and plaques typically located in the sun-protected areas of the body.^2^

The International Society of Cutaneous Lymphoma/European Organisation for Research and Treatment of Cancer (ISCL/EORTC) revised the TNMB (tumour, nodes, metastases, blood) staging system for MF and sezary Syndrome. According to the TNMB staging system, stage IA, IB, and IIA are considered early-stage MF.^3^ Skin-directed therapies such as topical corticosteroids, topical bexarotene, topical mechlorethamine hydrochloride, topical carmustine, electron-beam irradiation, narrowband UVB, and PUVA are used in early-stage MF.^4^ Among these treatments, topical corticosteroids are frequently used treatment agents in MF. They exhibit antineoplastic activity by inducing apoptosis in lymphocytes and inhibiting lymphocyte adhesion to endothelium and intercellular adhesion.^5^, ^6^ Besides, bexarotene is a selective retinoid X receptor agonist that exerts its antineoplastic effect via modulating cell proliferation, differentiation and apoptosis by regulating gene expression.^7^, ^8^ However, there are a limited number of studies comparing the efficacy, side effects, and safety profile of these treatment modalities in MF and treatment guidelines lack definitive recommendations regarding treatment modality preference.^9^

In the present study, the authors aimed to evaluate and compare the clinical efficacy, side effects and treatment compliance of topical 1% bexarotene gel and topical 0.05% clobetasol propionate ointment in patients with early-stage MF.

## Methods

### Participants and protocol

A retrospective chart review was carried out in the dermato-oncology unit of the department of dermatology in a tertiary care hospital. The study was approved by the local ethics committee (approval number: 3966). The study was performed in accordance with the latest version of the “Helsinki Declaration” and “Guidelines for Good Clinical Practice”.

Medical records of the patients with MF followed in the dermato-oncology outpatient clinic between September 2020 and September 2023 were compiled and analyzed. Patients with a definitive diagnosis of MF based on clinicopathological correlation and those under monotherapy were included in the study. The patients consisted of stage IA and IB MF patients, there was no folliculotropic MF in the patient group. Exclusion criteria were topical therapy for MF within the last month, systemic treatment within the last 2 months and oral retinoids within the last 4 months. Those who were pregnant, breastfeeding, or under 18-years of age were also not included in the study. Women of childbearing age were required to have a negative pregnancy test before initiation of the therapy. In addition, both men and women were informed about the need to use effective contraception methods throughout the treatment period.

Demographic and clinical characteristics of the patients such as age, sex, duration of the disease, clinical staging were recorded. TNMB classification system was used for staging the patients.^3^, ^10^ Patients with stage IA-IB MF were treated with 1% bexarotene gel twice daily (bexarotene group) and 0.05% clobetasol propionate ointment twice daily (clobetasole propionate group). Data of all patients were compiled after being followed for at least 2-years. Patients in both groups were treated for a minimum of 16-weeks. Patients were clinically assessed by the same dermatologist every four weeks. Clinical response was determined by using standard oncology criteria. Clinical Complete Response (CCR) (100% clear, a complete cutaneous remission), Partial Response (PR) (≥50% but <100% improvement), Stable Disease (SD) (<50% improvement or no change) and Progressive Disease (PD) (disease is worse than at baseline by ≥25%) were used to evaluate the clinical response.^11^ Additionally, clinical response time, relapse rates, time until relapse, and treatment compliance were noted. The presence of adverse effects, in the form of erythema, vesicles, crusts, hypo- or hyperpigmented areas, burning or itching sensations were recorded. In case of occurence of a treatment-limiting side effect, the dose interval was extended or the treatment was discontinued. In the case of SD or PD after at least 16-weeks of therapy, treatment was switched.

### Statistical analysis

All analyses were carried out using the IBM Statistical Package for the Social Sciences (SPSS) version 21.0. The normality distribution of continuous variables was checked with the Kolmogorov-Smirnov test. Data with nonparametric distribution were expressed as median (interquartile range) and categorical variables were expressed by number (percentage). Independent samples were compared with the Mann-Whitney *U* test and the Kruskal Wallis test. Pearson's Chi-Square and Fisher’s exact test were used for categorical variables. Two-sided p-values less than 0.05 were considered statistically significant.

## Results

A total of 40 patients with stage IA-IB MF were enrolled in the study. Of 40 patients, 20 patients (50%) were treated with 1% bexarotene gel and 20 patients (50%) were treated with 0.05% clobetasol propionate ointment. There were no substantial differences in age, gender ratio and clinical characteristics for each variable between the patient groups (p > 0.05) (Table 1).

**Table 1:** Demographic and clinical characteristics of the patients.

	Bexarotene Group (n = 20)	Clobetasol Propionate Group (n = 20)	p
Age, median (IQR), years	52.5 (21)	53.5 (16)	0.892
Sex, n (%)			0.749
Male	12 (60%)	11 (55%)	
Female	8 (40%)	9 (45%)	
Stage, n (%)			0.736
IA	13 (65%)	14 (70%)	
IB	7 (35%)	6 (30%)	
T, n (%)^a^			0.925
T1a	9 (45%)	11 (55%)	
T1b	4 (20%)	3 (15%)	
T2a	4 (20%)	3 (15%)	
T2b	3 (15%)	3 (15%)	
Disease duration, median (IQR), months	49 (19.5)	44.5 (48.75)	0.184
Prior Treatments, n (%)			
dbUVB	16 (80%)	15 (75%)	
Acitretin	1 (5%)	1 (5%)	
Naïve	3 (15%)	4 (20%)	

Data were expressed as median (interquartile range) in nonparametric continuous variables and n (%) in categoric variables.

Mann-Whitney *U* test, Pearson’s Chi-Square test, Fisher’s exact test and Kruskal Wallis test were used.

IQR, Interquartile Range.

a Based on Olsen et al.^12^

**Fig. 1:**
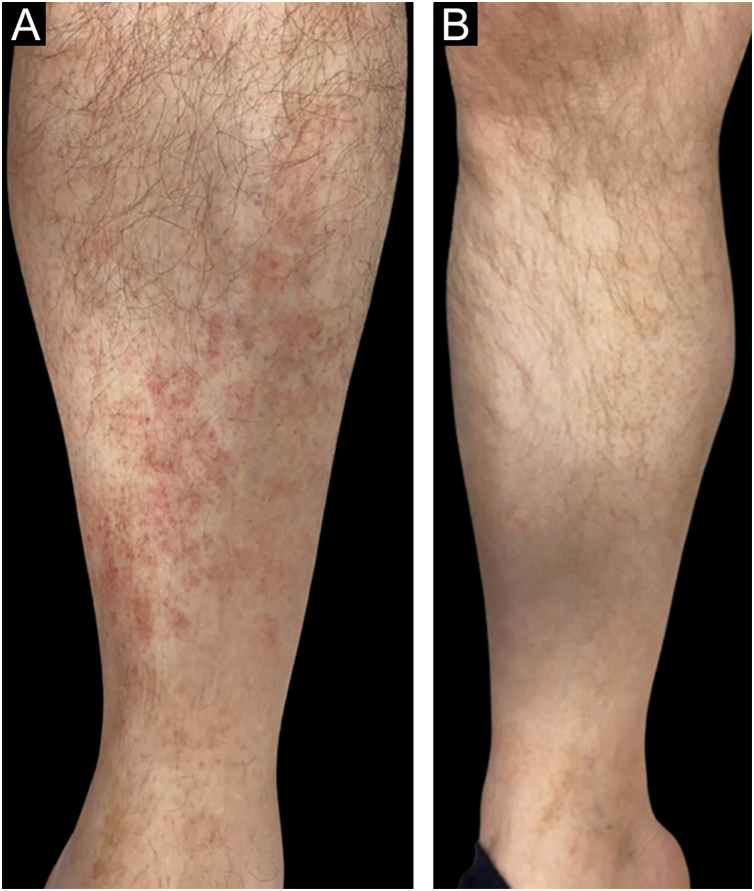
(A) Lesions on right leg in a 62-year-old man with stage IA MF. (B) Clinical complete response at week 16 of treatment with clobetasol propionate.

**Fig. 2:**
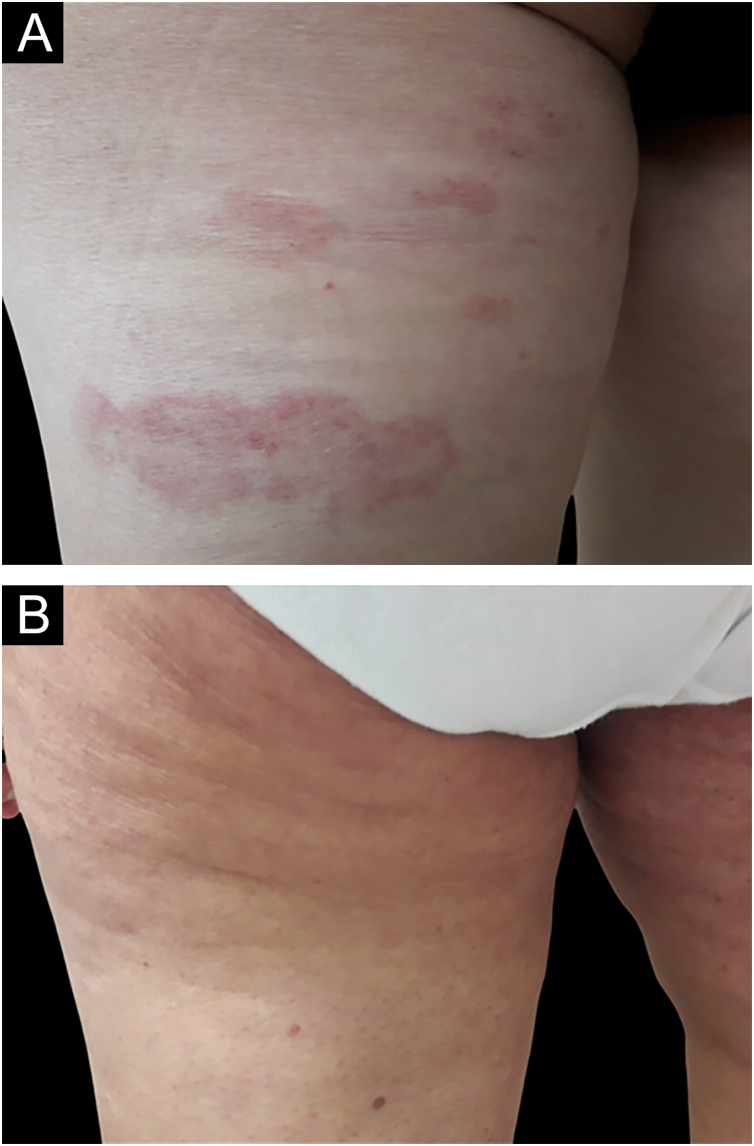
(A) Patch lesions on the left posterior thigh of a 55-year-old woman patient with stage IA MF. (B) Clinical complete response at week 16 of treatment with bexarotene.

In the bexarotene group, 11 patients (55%) had CCR, 5 patients (25%) had PR, and 4 patients (20%) had SD, while in the clobetasol propionate group, 10 patients (50%) had CCR, 9 patients (45%) had PR, and 1 patients (5%) had SD (Fig. 1, Fig. 2). PD was not observed in any treatment group. There was no statistically significant difference between the bexarotene and clobetasol propionate groups in terms of having CCR, PR and SD (p = 0.752, p = 0.185, p = 0.342, respectively) (Table 2).

**Table 2:** Comparison of clinical response, relapse rates, and treatment discontinuation in bexarotene and clobetasol propionate groups.

	Bexarotene Group (n = 20)	Clobetasol Propionate Group (n = 20)	p
Clinical Response, n (%)			
CCR	11 (55%)	10 (50%)	0.752
PR	5 (25%)	9 (45%)	0.185
SD	4 (20%)	1 (5%)	0.342
Time to achieve CCR, median (IQR), months	3 (3)	2 (3)	0.512
Time to achieve PR, median (IQR), months	3 (2)	3 (4)	0.784
Relapse^a^, n (%)	6 (54.5%)	7 (70%)	0.659
Duration of Remission/ Time Until Relapse, median (IQR), months	10.5 (7.75)	4 (6)	0.032*
Irritation, n (%)	11 (55%)	0	0.001^a^
Discontinuation of Treatment, n (%)	8 (40%)	9 (45%)	0.749
Due to Irritation, n (%)	3 (15%)	0	0.231
Due to Inadequate Response, n (%)	5 (25%)	9 (45%)	0.185

Data were expressed as median (interquartile range) in nonparametric continuous variables and n (%) in categoric variables.

Mann-Whitney *U* test, Pearson’s Chi-Square test, and Fisher’s exact test were used.

CCR, Clinical Complete Remission; IQR, Interquartile Range; PR, Partial Response; SD, Stable Disease.

* p < 0.05.

a Relapse rate was evaluated only in patients who achieved CCR.

The median time required to achieve CCR was 3-months (IQR:3) in the bexarotene group and 2-months (IQR:3) in the clobetasol propionate group. Median time required to achieve PR was 3-months (IQR:2) in the bexarotene group and 3-months (IQR:4) in the clobetasol propionate group. There was no statistically significant difference between the bexarotene and clobetasol propionate groups in terms of the required time to achieve CCR and PR (p = 0.512, p = 0.784, respectively) (Table 2).

The relapse rate was evaluated only in patients who achieved CCR. Relapse occurred in 6 (54.5%) of 11 patients who achieved CCR in the bexarotene group and in 7 (70%) of 10 patients who achieved CCR in the clobetasol propionate group. There was no statistically significant difference between the bexarotene and clobetasol propionate groups in terms of relapse (p = 0.659) (Table 2).

**Fig. 3:**
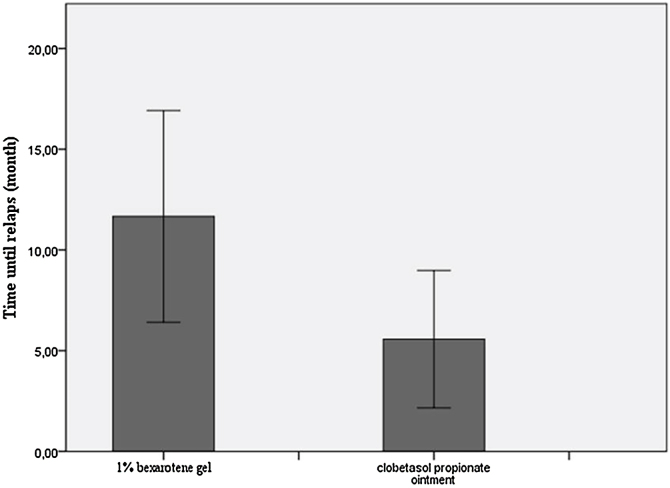
Comparison of time until relaps between the bexarotene group and the clobetasol propionate group.

The median duration of remission/Median time until relapse was 10.5-months (IQR:7.75) in the bexarotene group and 4-months (IQR:6) in the clobetasol propionate group. The remission period was statistically significantly longer in the bexarotene group (p = 0.032) (Table 2, Fig. 3).

Irritation symptoms such as burning, stinging, itching, erythema, and eczematous lesions were seen only in the bexarotene group. Eleven out of 20 patients (55%) in the bexarotene group experienced irritation symptoms throughout the treatment period (p = 0.001). Signs of irritation were confined to the cutaneous areas where bexarotene gel was applied. Cutaneous side effects of topical corticosteroids such as atrophy, hypo-hyperpigmentation, and striae were not seen in the clobetasol propionate group. There was no difference between groups in terms of treatment compliance (p = 0.749). In the bexarotene group, 3 (15%) patients discontinued treatment due to irritation and 5 (25%) patients due to inadequate response whereas in the clobatesol propionate group 9 (45%) patients discontinued treatment due to inadequate response (Table 2).

When patients with MF were evaluated further according to T stage based on the presence of patches and patches/plaques,^12^ there was no statistically significant difference in terms of CCR, PR and SD between T1a vs. T1b and T2a vs. T2b groups in both the bexarotene group and the clobetasol propionate group (CCR p = 1, PR p = 1, SD p = 1 for T1a vs. T1b in the bexarotene group; CCR p = 1, PR p = 1, SD p = 1 for T2a vs. T2b in the bexarotene group; CCR p = 0.209, PR p = 1, SD p = 0.214 for T1a vs. T1b in the clobetasol propionate group; CCR p = 0.40, PR p = 0.40 for T2a vs. T2b in the clobetasol propionate group).

## Discussion

Mycosis fungoides is a disease that requires long-term treatment and follow-up after diagnosis. Topical agents and skin-directed therapies are preferred in the first-line treatment of early-stage MF for two main reasons.^9^ Firstly, effective results can be achieved by skin-directed therapies minimizing the toxic side effects. Furthermore, aggressive treatments have not been shown to improve survival in MF.^13^ However, these options may not always be available depending on hospital facilities and place of residence and/or switching to another treatment may be required due to side effects, inadequate clinical response, and refractory disease.

In this study, the authors evaluated the clinical efficacy of topical bexarotene and clobetasol propionate in 40 patients with stage IA and IB MF. There was no difference in clinical efficacy and relapse rate between bexarotene and clobetasol propionate groups. The remission period was significantly longer in the bexarotene group. On the other hand, irritation symptoms were significantly higher in the bexarotene group. To the best of our knowledge, this is the first study comparing the efficacy and tolerability of topical bexarotene and clobetasol propionate in the treatment of MF.

There are a limited number of studies investigating the effectiveness of topical bexarotene or clobetasol propionate in the literature. In a preliminary study, the safety and effectiveness of two topical retinoid gels (9-cis retinoic acid vs. bexarotene) were evaluated in 6 patients with stage I CTCL. Of the 18 index lesions, complete response was achieved in 56%, and PR was achieved in 22% in the bexarotene group.^14^ In the present study, CCR and PR rates in the bexarotene group were similar to the findings of this preliminary study (CCR: 55%, PR: 25%, respectively). Whereas on the side of clobetasol propionate, Zackheim et al.^15^ conducted a prospective study to determine the effectiveness of topical corticosteroids in MF with 79 (51 T1, 28 T2) patients. Patients applied topical class I to III corticosteroids twice daily. Of 51 stage T1 patients, 63% achieved complete remission, 31% achieved PR, total response rate (CCR + PR) was 94%. Of 28 stage T2 patients, 25% achieved complete remission, 57% achieved PR, total response rate (CCR + PR) was 82%.^15^ In the present study, patients with T1, 2 MF had 50% CCR and 45% PR, and the total response rate was 95% which was in line with the findings of the study by Zackheim et al.^15^

In phase I and phase II trials of bexarotene gel, 67 patients with early-stage MF (41 IA, 20 IB, 5 IIA, 1 IIB) were included. Of 67 patients, 21% of patients achieved CCR and 42% of patients achieved PR whereas 21% of patients had SD and 16% of patients had PD according to Physician’s Global Assessment (PGA). The median projected time to achieve CRR and PR was 20.1-weeks and the median projected duration of response from the start of therapy was 99-weeks. The median durability of response (from the onset of response to relapse) was 61.1-weeks.^11^ In this study, 55% of the patients achieved CCR in the bexarotene group and it was found to be higher compared with the phase I/II trial. Possible reasons for this difference can be listed as follows: (i) In this study, there were only stage IA and IB patients. The authors did not include stage IIA and IIB patients. (ii) In the phase I/II trial, 35.8% of patients were refractory or intolerant to previous treatments. However, there were no refractory or intolerant patients in the study group. While the median remission period of 14 patients who achieved CCR in the phase I/II trial was stated as 8 weeks, the median remission duration of patients who achieved CCR was 10.5 months in this study. Similarly, the difference between remission periods can be explained by the fact that the authors do not have advanced-stage or refractory patients. Additionally, in the phase I/II trial, 52% of 42 treatment-responsive patients continued treatment with 1% bexarotene gel, while 31% used 0.5% gel and 17% used 0.1% gel. It can be suggested that this difference in drug concentrations may also affect the rates and duration of remission.

In phase III trial of 1% bexarotene gel, 50 patients with refractory or persistent early-stage CTCL (25 IA, 22 IB, 2 IIA, 1 IIB) were included. Of 50 patients, 2% patients achieved CCR and 42% of patients achieved PR whereas 36% patients had SD and 16% of patients had PD according to PGA.^16^ When the findings of phase III trial was evaluated in comparison with the present study, in the phase III trial, the CCR rate with bexarotene was lower, while the PR and SD rates were higher, and 16% of the patients had PD. But unlike this study, refractory or persistent patients were included in the phase III trial, and there were also stage IIA and IIB patients. As a result of the phase III trial, stage IIA and IIB patients were reported to be unresponsive to treatment. The presence of refractory and advanced stage patients in the phase III trial might have caused lower CCR rates.

In the present study, there were other significant findings. In the patient group achieving CCR, median time until relapse was 10.5-months in the bexarotene group and 4-months in the clobetasol propionate group. The median time until relapse with bexarotene was significantly longer than the clobetasol propionate group. Irritation symptoms were observed in 55% of the patients in the bexarotene group and 3 (15%) patients discontinued treatment due to irritation. In the phase I/II trial, 4 (6%) patients discontinued bexarotene gel, 3 of them due to cutaneous side effects and 1 of them due to trigeminal neuralgia.^11^ In the phase III trial, out of 50 patients, 94% of patients experienced at least 1 treatment-related adverse effect and 26% of them experienced moderately severe and severe adverse effects. Overall, the most common adverse effect was irritant dermatitis.^16^ In the present study, irritation symptoms were also the most common side effect related to bexarotene which was in line with the findings of phase studies.

There are several limitations of the present study. The main limitation of this study was retrospective design. In addition, stage IIA patients were not included in this study despite being classified in the early-stage category because phototherapy and systemic treatments were preferred primarily in the management of these patients in the clinical approach.

## Conclusion

In conclusion, both topical 1% bexarotene gel and topical 0.05% clobetasol propionate ointment were found to be effective and well-tolerated options in early-stage MF. Although irritation symptoms due to topical bexarotene were significantly more common than topical clobetasol propionate, it did not affect compliance with treatment. Considering the easy accessibility, cost-effectiveness, and limited and manageable cutaneous side effects of corticosteroids, it can be suggested that topical corticosteroids will be ahead of topical bexarotene in the treatment algorithm of early-stage MF. However, topical bexarotene is also an effective alternative treatment option with a longer remission period. Further prospective randomized controlled studies with a greater number of patients are needed to fully address the efficacy and safety profile of skin-directed treatment options in MF.

## Financial support

None declared.

## Authors’ contributions

Aslı Aksu Çerman: The study concept and design; data collection, or analysis and interpretation of data; statistical analysis; writing of the manuscript or critical review of important intellectual content; data collection, analysis, and interpretation; effective participation in the research guidance; intellectual participation in the propaedeutic and/or therapeutic conduct of the studied cases; critical review of the literature; final approval of the final version of the manuscript.

Pinar Ozdemir Cetinkaya: Writing of the manuscript or critical review of important intellectual content critical review of the literature; final approval of the final version of the manuscript.

Birgul Ozkesici Kurt: Writing of the manuscript or critical review of important intellectual content critical review of the literature; final approval of the final version of the manuscript.

Artun Kırker: Data collection; final approval of the final version of the manuscript.

İlknur Altunay: Final approval of the final version of the manuscript.

## Conflicts of interest

None declared.
